# Valorization of fruit pomaces for glycosidic enzymes production via solid state fermentation

**DOI:** 10.1038/s41598-026-52343-8

**Published:** 2026-05-19

**Authors:** Zahraa H. Hafez, Abeer E. Mahmoud, Hadeer A. Mahmoud, Amira T. Mohammed, Mohamed K. Ibrahim

**Affiliations:** 1 Biochemistry Department, Biotechnology Research Institute, National Research Centre, Cairo, Egypt; 2 Microbiology Department, Faculty of Science, Ain Shams University, Cairo, Egypt

**Keywords:** Agro-industrial Wastes, Glycosidases, Pomegranate Peels, *Candida guilliermondii*, Amylase, RSM, Circular bioeconomy., Biochemistry, Biological techniques, Biotechnology, Microbiology, Plant sciences

## Abstract

Agro-industrial fruit pomaces represent complex, nutrient-rich substrates that can support microbial enzymes production within circular bioeconomy frameworks. This study systematically compared grape, mango, orange, and pomegranate pomaces as solid substrates for glycosidic enzymes production (amylase, xylanase, pectinase) using 14 microbial strains under solid-state fermentation conditions with the aim of identifying an efficient microorganism–substrate system that produces the highest glycosidic enzyme activity. For the 14 strains studied, *Candida guilliermondii* NRRL Y-2075 yielded the highest reported amylase activity (4344.67 U/gds) when cultivated on pomegranate pomace with no detectable activity in the unfermented pomace. Response surface methodology (RSM) based on a central composite design was subsequently applied to identify the optimal operational region for amylase production by evaluating pH, inoculum size, incubation temperature and time. Maximum amylase activity (4839.05 U/gds) was obtained at pH 5.6, 12.2% inoculum size, 30.7 °C incubation temperature, and 24 h of incubation. Experimental validation closely matched model predictions. Additional one-factor-at-a-time experiments demonstrated that supplementation with external carbon, nitrogen, amino acids, or metal ions did not enhance enzyme production, indicating that pomegranate pomace alone provides sufficient nutrients for efficient amylase synthesis. Collectively, the results suggest that pomegranate pomace can function as a nutritionally sufficient SSF substrate, reducing process complexity and supplementation requirements for sustainable amylase production.

## Introduction

 Agro-industrial activities generate over 2 billion tons of waste annually, primarily from agricultural production and food processing industries^1,2^. Improper disposal of this organic waste through landfilling or incineration contributes to serious environmental problems, including greenhouse gas emissions, unpleasant odors, and contamination of water and soil^3^.

Recently, agro-industrial waste, specifically by-products from fruit processing, has been suggested as a potential source of raw materials, rather than as an environmental burden. These wastes contain large amounts of bioactive compounds, which serve as an excellent substrate for microbial growth with a potential of high-value metabolites production, including enzymes, pigments, and single-cell proteins. Furthermore, many industries, like pharmaceutical, textile, and food industries, show an increasing reliance on microbial metabolites. Agro-industrial waste valorization allows not only the incorporation of zero waste and circular economy principles but also advances Sustainable Development Goals 12 and 13^4,5^.

Solid-state fermentation (SSF) is one of the novel and effective eco-technological ways of converting agro-industrial waste into products of value^6^. Per the definition, SSF is defined as microbial cultivation on solid material with little to no free water. Not only is SSF a unique method of continuously producing value-added products, but it is also one of the most energy-efficient and environmentally sustainable ways of recovering value from agro-industrial waste by utilizing an organic substrate^7,8^.

Glycoside hydrolases (GHs) are enzymes that hydrolyze glycosidic bonds in carbohydrates to soluble sugars^9^. They are commonly referred to as glycosidases, and occur in almost all living organisms, where they have various biological roles^10^. GHs have numerous industrial applications, including biofuel production and the paper industry, where they hydrolyze starch coatings to enhance paper smoothness, improving writing quality^11^.

Among GHs, amylases are widely utilized across various industries. Within the detergent industry, amylases are used to remove starchy stains, while in the textile industry, they are used to desize fabrics. They are also very important in the food industry, as they are essential enzymes in baking, brewing, and starch liquefaction. Amylases also have diagnostic and therapeutic applications in the clinical and pharmaceutical sectors^12,13^. However, there is limited research exploring the comparative potential of diverse fruit wastes under SSF conditions for glycosidic enzyme production. Additionally, the optimization of this process using Response Surface Methodology (RSM), a statistical technique ideal for maximizing enzyme production by adjusting multiple fermentation parameters, remains underexplored.

Accordingly, this study was designed to (i) comparatively evaluate different fruit pomaces as SSF substrates for glycosidic enzyme production, (ii) identify the most efficient microorganism–substrate combination, (iii) define the dominant process variables controlling amylase production using statistically guided RSM, and (iv) assess whether external supplementation provides additional benefits beyond the intrinsic nutritional capacity of the selected pomace. This sequential approach links substrate selection, process optimization, and biological interpretation within a unified bioprocess development framework.

## Materials and methods

### Fruit pomace

Fruit pomaces, including pomegranate (*Punica granatum*) peels, mango (*Mangifera indica*) peels, orange (*Citrus sinensis*) peels and grape (*Vitis vinifera*) pomace, were generously collected from juice extraction shops and food processing factories located in Cairo, Egypt, during their respective harvesting seasons. Pomaces were collected fresh, washed with tap water, minced in a mixer and stored at − 20 ℃ until used. The chemical composition of the fruit pomaces used in this study was adopted from a previously published work conducted in our laboratory, in which proximate analysis was carried out according to AOAC methods and the C/N ratio was indirectly estimated by using total carbohydrate content as the carbon source and crude protein content as the nitrogen source^14^. The data are presented in Table 1.

**Table 1:** Proximate composition of different fruit pomaces.

Parameter	Pomegranate pomace	Orange pomace	Grape pomace	Mango pomace
Moisture (%)	70.93 ± 0.005	69.57 ± 0.089	71.64 ± 0.023	68.57 ± 0.087
Organic matter (%)	97.05 ± 0.025	93.81 ± 0.00	96.93 ± 0.026	97.24 ± 0.023
Crude protein (%)	8.31 ± 0.107	13.90 ± 0.058	13.23 ± 0.268	10.59 ± 0.159
Crude fiber (%)	17.12 ± 0.023	10.95 ± 0.049	13.40 ± 0.023	6.14 ± 0.003
Fat content (%)	1.44 ± 0.219	5.17 ± 0.065	5.82 ± 0.079	1.74 ± 0.03
Carbohydrate content (%)	70.18 ± 0.173	63.79 ± 0.07	64.48 ± 0.289	78.77 ± 0.176
C/N ratio	8.44	4.59	4.87	7.44

Values are expressed as the mean ± standard error.

### Microorganisms

Bacterial strains were obtained from the Molecular Genetics Department, Biotechnology Research Institute, National Research Centre. One gram of various soil samples collected from different locations in Egypt were transported to the microbial genetics laboratory and transferred into fresh 100 mL salt medium [(g/L): glucose, 10; NaNO₃, 0.5; K₂HPO₄, 1.0; MgSO₄·7 H₂O, 0.5; KCl, 0.5; FeSO₄·7 H₂O, 0.001]. The cultures were incubated at 37 °C for 48 h and the bacterial strains were identified biochemically and morphologically according to Holt et al.^15^. Molecular identification was subsequently performed by 16 S rDNA gene sequencing^16^(Table 2). Yeast strains were purchased from the Agricultural Research Service, Peoria, Illinois, USA (Table 3).

**Table 2:** Bacterial strains used during the study.

Strain Number	Strain name	Accession number
1	*Bacillus cereus*	LC315566
2	*B. subtilis*	LC315565
3	*B. licheniformis*	LC315920
4	*B. thuringiensis*	LC438914
5	*B. amyloliquefaciens*	PV569636
6	*B. proteolyticus*	PV569637
7	*B. velezensis*	PV569638
8	*B. siamensis*	PV569639
9	*B. atrophaeus*	PV569640
10	*B. amyloliquefaciens plantarum*	PV569641

**Table 3:** Yeast strains used during the study.

Strain number	Strain name	Accession number
1	*Kluyveromyces marxianus*	NRRL Y-7571
2	*Kluyveromyces marxianus*	NRRL Y-8281
3	*Candida bambicola*	NRRL Y-17,069
4	*Candida guilliermondii*	NRRL Y-2075

### Medium composition and growth condition

Bacterial strains were adapted according to the method described by the American Public Health Association^17^, while yeast strains were adapted following the procedure outlined by Wickerham^18^.

### Screening of different microorganisms for glycosidases production

Ten bacterial strains as well as four yeast strains were screened to utilize various fruit pomaces wastes, including grape and mango, orange, and pomegranate, to produce key glycosidic enzymes, namely amylase, xylanase, and pectinase under SSF. The strain demonstrating the highest overall enzyme activity was selected for subsequent optimization of fermentation parameters.

### Solid state fermentation

For SSF, suspension aliquots of 1 mL (approximately 1.5 × 10^8^ CFU/ mL, corresponding to a 0.5 McFarland standard) were inoculated into 250 mL Erlenmeyer flasks containing 10 g of sterilized fruit pomace, which was autoclaved at 121 °C for 20 min at 15 psi using an autoclave sterilizer (Tomy, SX-700, Tokyo, Japan). Unfermented (non-inoculated) pomace samples were prepared in parallel under identical sterilization and incubation conditions and served as controls. All flasks were incubated statically at 35 °C for 48 h using an incubator shaker (Thermo Fisher Scientific, MAXQ 481R HP, Massachusetts, USA)^19^.

### Enzyme extraction

Crude enzyme was extracted from the fermented fruit pomaces by adding distilled water at a 1:10 (w/v) ratio and shaking (150 rpm) at 25 °C for 60 min using an incubator shaker (Thermo Fisher Scientific, MAXQ 481R HP, Massachusetts, USA). The resulting slurry was then filtered through a double-layered muslin cloth by manual squeezing, and the filtrate was collected and used as the crude enzyme extract^20^.

### Enzyme assay

Glycosidases activities were determined spectrophotometrically by measuring reducing sugars released according to Nelson^21^ and Somogyi^22^ at 540 nm using a UV–Vis spectrophotometer (Agilent Technologies, Cary 100, California, USA). One unit of enzyme activity was defined as the amount of enzyme that releases µmol equivalents of reducing sugars (maltose for amylase, xylose for xylanase, and galacturonic acid for pectinase) in 1 min under assay conditions. Enzyme activities are expressed as unit per gram dry substrate (U/gds). For each assay, a corresponding sample control (zero-time control) was performed by immediately terminating the reaction upon enzyme addition. The control values were subtracted from the test readings to account for pre-existing reducing sugars and non-enzymatic background.

$$\:\mathrm{U}\:/\:\mathrm{m}\mathrm{L}\:=\:\frac{\mathrm{O}.\:\mathrm{D}\:\mathrm{o}\mathrm{f}\:\mathrm{t}\mathrm{e}\mathrm{s}\mathrm{t}}{\mathrm{O}.\:\mathrm{D}\:\mathrm{o}\mathrm{f}\:\mathrm{s}\mathrm{t}\mathrm{a}\mathrm{n}\mathrm{d}\mathrm{a}\mathrm{r}\mathrm{d}}\times\:\frac{\mathrm{C}\mathrm{o}\mathrm{n}\mathrm{c}.\:\:\mathrm{o}\mathrm{f}\:\mathrm{s}\mathrm{t}\mathrm{a}\mathrm{n}\mathrm{d}\mathrm{a}\mathrm{r}\mathrm{d}}{\mathrm{M}\mathrm{o}\mathrm{l}\mathrm{e}\mathrm{c}\mathrm{u}\mathrm{l}\mathrm{a}\mathrm{r}\:\mathrm{w}\mathrm{e}\mathrm{i}\mathrm{g}\mathrm{h}\mathrm{t}\:\mathrm{o}\mathrm{f}\:\mathrm{s}\mathrm{t}\mathrm{a}\mathrm{n}\mathrm{d}\mathrm{a}\mathrm{r}\mathrm{d}}\times\:\frac{1}{\mathrm{m}\mathrm{L}\:\mathrm{o}\mathrm{f}\:\mathrm{e}\mathrm{n}\mathrm{z}\mathrm{y}\mathrm{m}\mathrm{e}}$$

### Optimization of fermentation process parameters

Various physico-chemical and nutritional parameters influencing enzyme production during SSF were optimized. Optimization was performed using response surface methodology with a central composite design model for four quantitative factors, while qualitative factors were subsequently optimized using the one-factor-at-a-time (OFAT) approach.

### Response surface methodology based on central composite design optimization

The optimization of four independent variables, namely pH (A), inoculum size (B), incubation temperature (C), and incubation time (D), was performed using a CCD model constructed with Design-Expert software (version 11.1.2.0, Stat-Ease Inc., Minneapolis, MN, USA; https://www.statease.com/software/design-expert). The experimental design was based on three levels and two axial points for each factor, where the coded values of -1, 0, and + 1 represent the low, middle, and high levels, respectively. The CCD model consisted of 8 axial points, 10 center points, and two replicates of the factorial points, resulting in a total of 50 experimental runs. The design parameters and their corresponding coded and actual values are summarized in Table 4.

**Table 4:** Experimental factors and their corresponding coded and actual values used in the CCD model.

Factor	Name	Units	Min.	Max.	Coded Low (-1)	Coded High (+ 1)	Mean (0)	Std. Dev.
A	pH	-	3.00	12.00	-1 = 5.61	+ 1 = 9.39	7.50	1.78
B	Inoculum size	%	5.00	30.00	-1 = 12.24	+ 1 = 22.76	17.50	4.94
C	Temp.	°C	25.00	45.00	-1 = 30.80	+ 1 = 39.20	35.00	3.95
D	Time	h	7. 46	64.54	-1 = 24.00	+ 1 = 48.00	36.00	11.28

The effect of the interaction of various fermentation process parameters on the amylase production (Z axis) was studied by plotting three-dimensional response surface curves against any two independent variables while keeping the other independent variable at their (0) levels. Therefore, six response surfaces were obtained by considering all the possible combinations.

### Validation of response surface methodology optimum conditions

The optimized conditions obtained from the RSM model were validated by conducting independent experiments in triplicate under the predicted optimal parameters. Amylase activity (U/gds) was measured as the response variable.

### Statistical description of the RSM model

Data were analyzed using Design-Expert software (version 11.1.2.0, Stat-Ease Inc., Minneapolis, MN, USA; https://www.statease.com/software/design-expert). Model adequacy was evaluated by analysis of variance (ANOVA). The significance of model terms was assessed, and the model fit was determined using R², adjusted R², predicted R², and lack-of-fit tests. Three-dimensional response surface plots were generated to visualize interaction effects between the independent variables.

### One-factor-at-a-time optimization

The influence of various qualitative factors was evaluated using the one-factor-at-a-time (OFAT) approach. The effects of different carbon sources, nitrogen sources, amino acids and metal ions were tested under optimized CCD conditions to further enhance enzyme production.

### Effect of different carbon sources

To examine the effect of additional carbon sources on enzyme production, the fermentation medium was supplemented individually with glucose, xylose, fructose, mannose, sucrose, lactose, sorbitol, mannitol, and soluble starch at a 1% (w/w) concentration.

### Effect of different nitrogen sources

To study the effect of supplementation of additional nitrogen sources on enzyme production, some organic nitrogen sources (malt extract, peptone, urea, yeast extract, and casein) and inorganic nitrogen sources (ammonium sulfate and ammonium nitrate) were added solely to the fermentation medium at 1% (w/w) concentration on an equivalent nitrogen basis.

### Effect of different amino acids

The effect of adding different amino acids to the fermentation medium was studied. Arginine, asparagine, glycine, histidine, methionine, leucine, tyrosine, and tryptophan were added solely to the fermentation medium at a concentration of 1% (w/w).

### Effect of various metal ions

Various metal chlorides, namely calcium, barium, cobalt, copper, ferric, ferrous, magnesium, potassium, sodium, and zinc were separately added to the fermentation medium at a concentration of 1% (w/w) to investigate their effect on enzyme production.

### Statistical analysis

All data are expressed as mean ± standard deviation (SD) based on three independent batches. Statistical analyses were performed using SPSS version 16.0 (SPSS Inc., Chicago, IL, USA). A one-way ANOVA, followed by Tukey’s HSD post hoc test, was conducted to determine significant differences among treatment means. Differences were considered statistically significant at *p <* 0.05.

## Results and discussion

The global demand for industrial enzymes has increased significantly over recent decades. The market value of industrial enzymes grew from approximately 0.31 billion USD in 1960 to 6 billion USD in 2020, and it is projected to surpass 9 billion USD by 2027^23^. Microbial enzymes are favored for their thermal and pH stability, ease of cultivation, and multifunctional applications, making them highly suitable for diverse industrial applications^24^. Therefore, our study focused on producing various glycosidases using different microorganisms under SSF.

### Screening and Identification of the most potent Glycosidase-producing microorganism

The screening of 14 microorganisms for glycosidases production (amylase, pectinase, and xylanase) using different fruit pomaces (mango, orange, pomegranate and grape) as substrates revealed significant variations in enzymatic activity, highlighting the influence of microbial species and substrate composition on enzyme yield^25^. This study provides valuable insights into the potential of agro-industrial byproducts as sustainable substrates for microbial enzyme production.

**Fig. 1:**
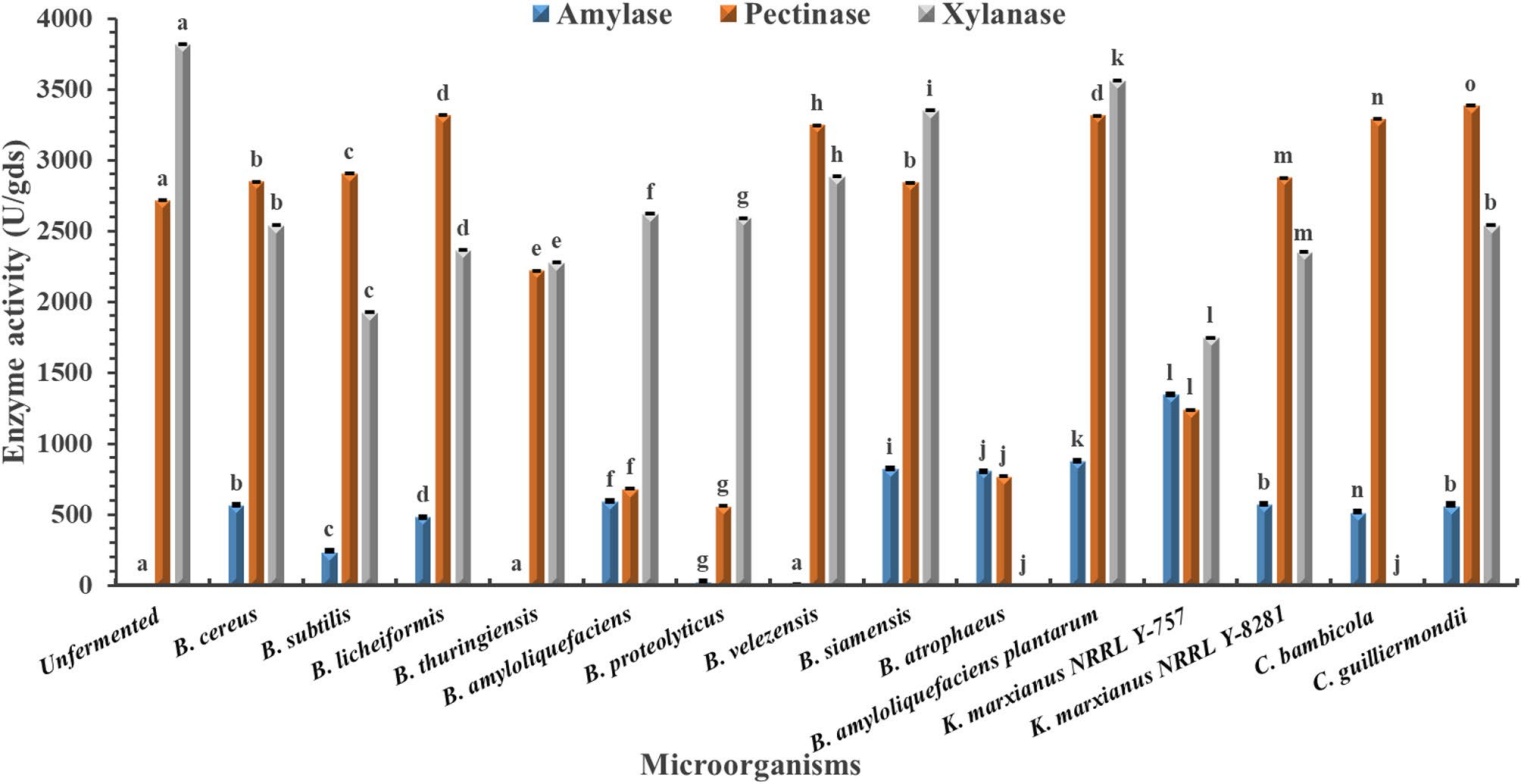
Amylase, pectinase, and xylanase activities of different microbes cultivated on grape waste under solid-state fermentation. Unfermented fruit pomace: non-inoculated autoclaved fruit pomace. Data are presented as mean ± standard deviation from three independent batches. Differences between groups were analyzed using one-way ANOVA/Tukey HSD post hoc tests. Means bearing different letters superscripts within the same enzyme are significantly different from each other, at a significance level of *p* < 0.05.

**Fig. 2:**
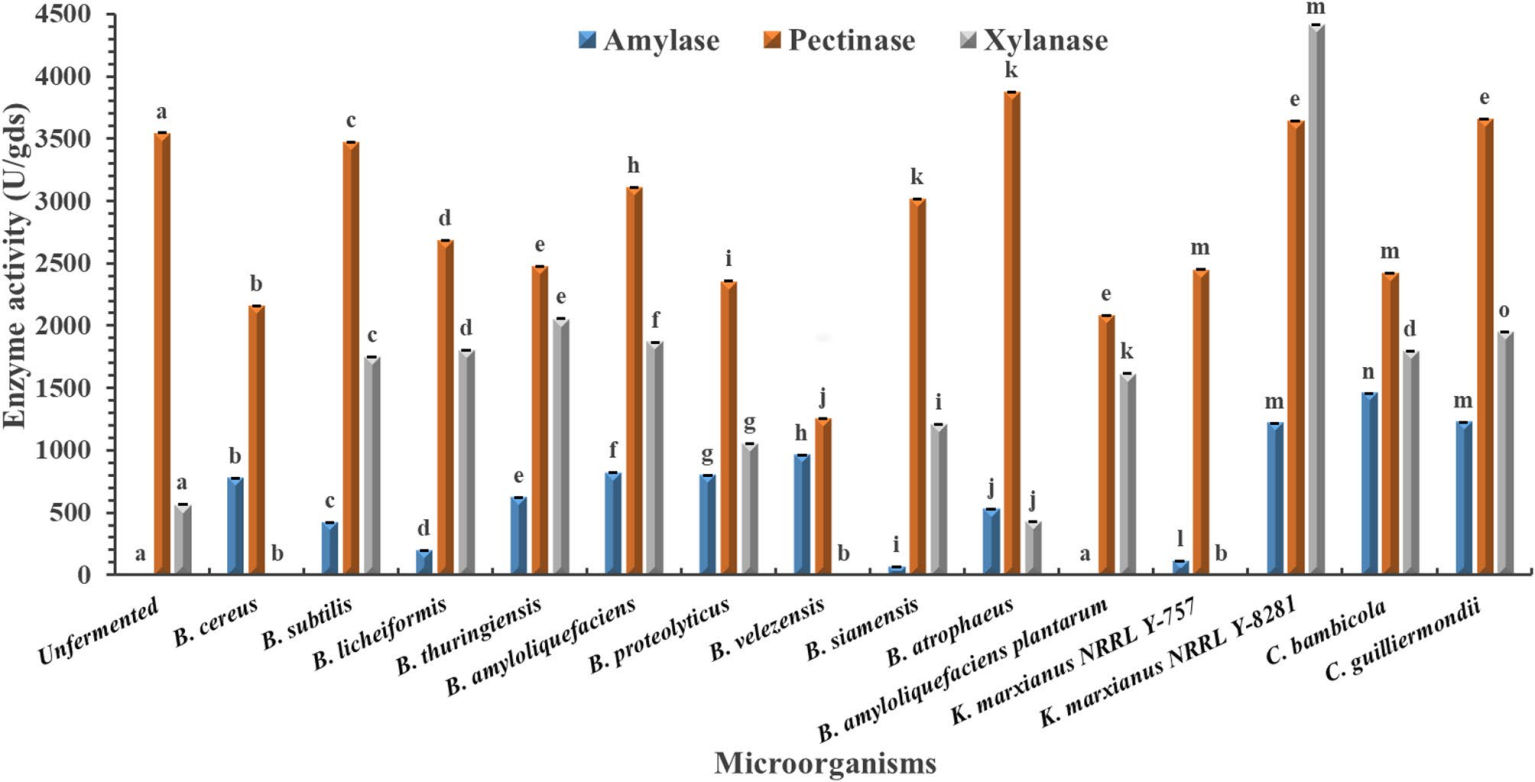
Amylase, pectinase, and xylanase activities of different microbes cultivated on mango waste under solid-state fermentation. Unfermented fruit pomace: non-inoculated autoclaved fruit pomace. Data are presented as mean ± standard deviation from three independent batches. Differences between groups were analyzed using one-way ANOVA/Tukey HSD post hoc tests. Means bearing different letters superscripts within the same enzyme are significantly different from each other, at a significance level of *p* < 0.05.

**Fig. 3:**
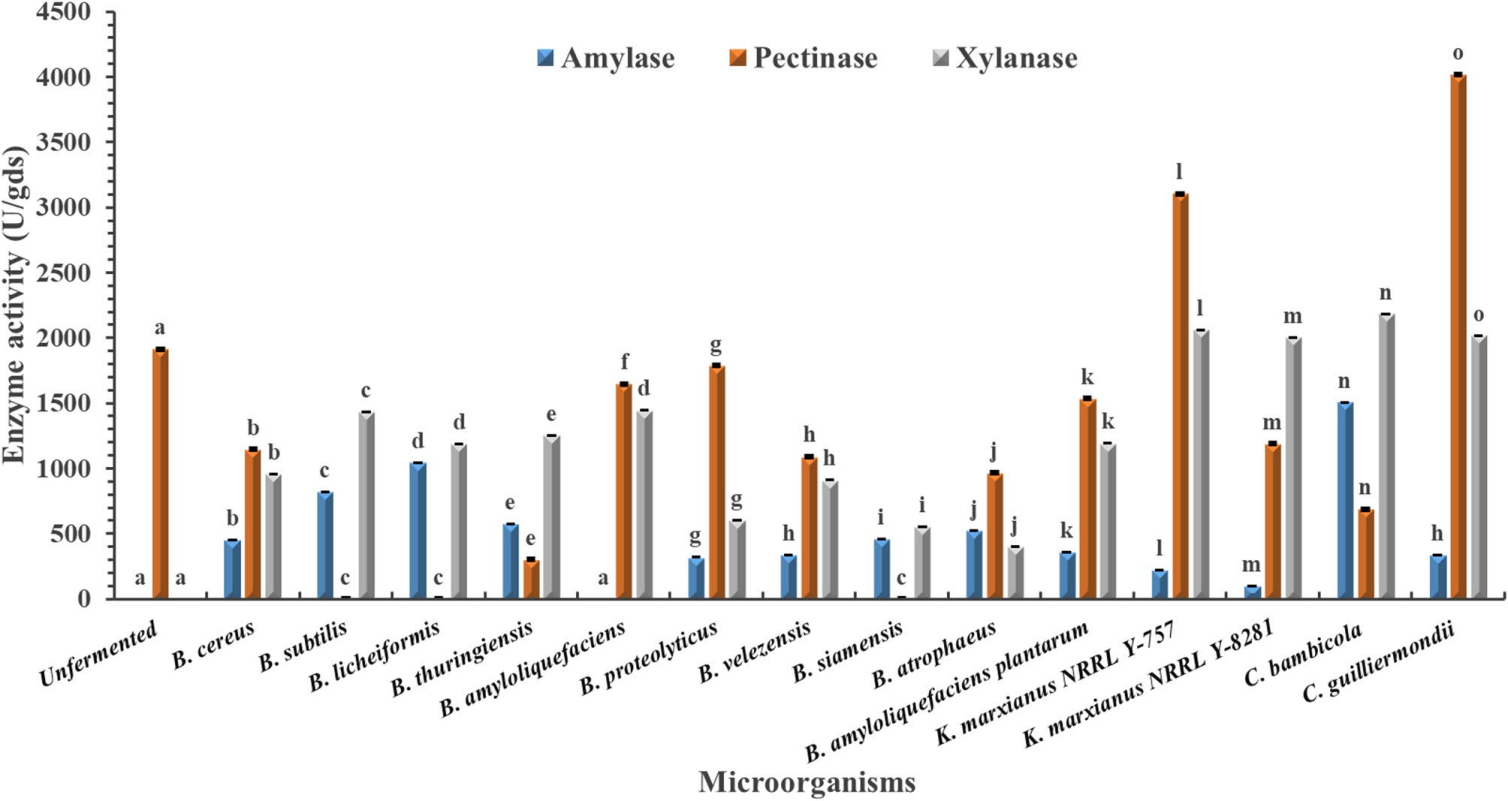
Amylase, pectinase, and xylanase activities of different microbes cultivated on orange waste under solid-state fermentation. Unfermented fruit pomace: non-inoculated autoclaved fruit pomace. Data are presented as mean ± standard deviation from three independent batches. Differences between groups were analyzed using one-way ANOVA/Tukey HSD post hoc tests. Means bearing different letters superscripts within the same enzyme are significantly different from each other, at a significance level of *p* < 0.05.

**Fig. 4:**
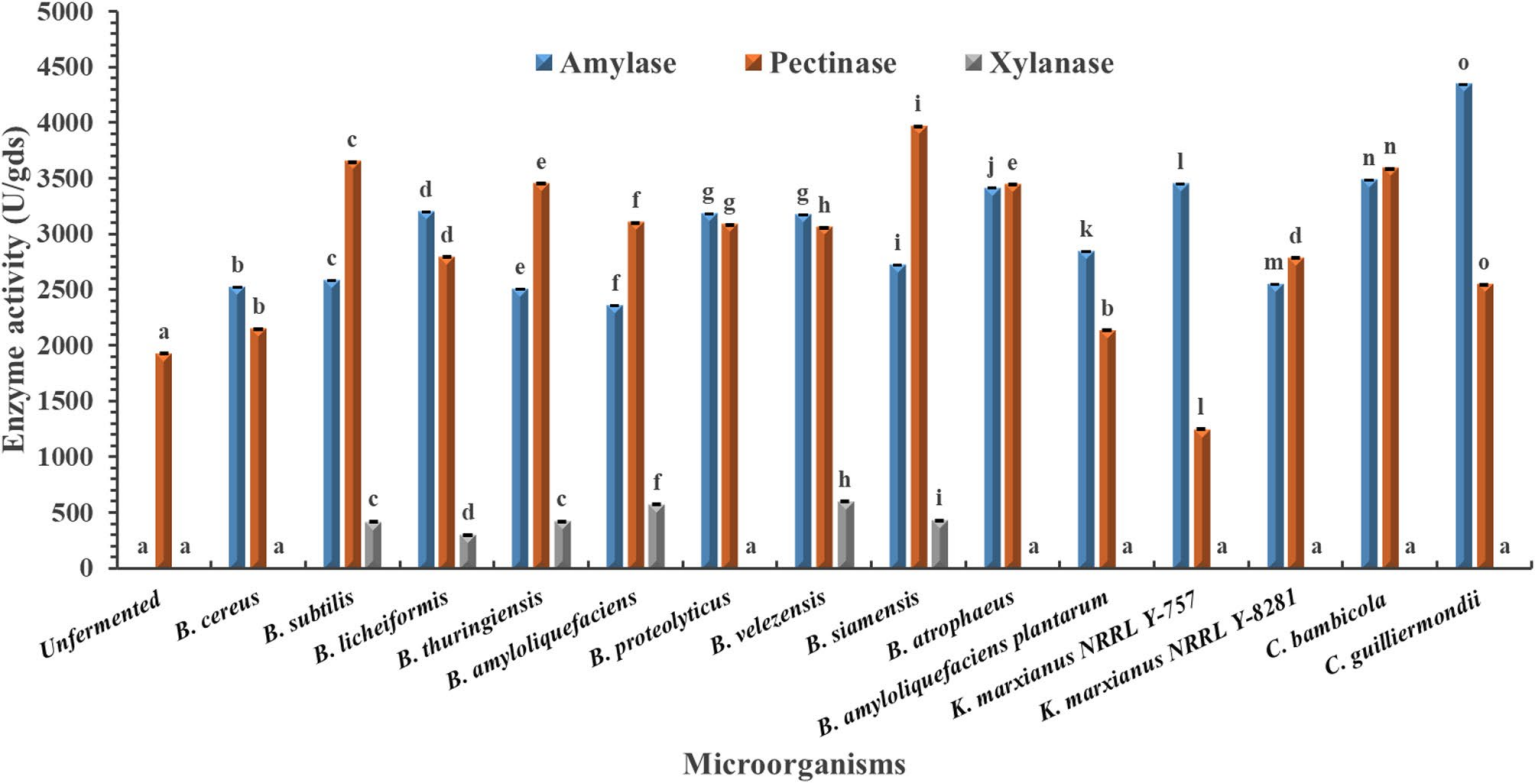
Amylase, pectinase, and xylanase activities of different microbes cultivated on pomegranate waste under solid-state fermentation. Unfermented fruit pomace: non-inoculated autoclaved fruit pomace. Data are presented as mean ± standard deviation from three independent batches. Differences between groups were analyzed using one-way ANOVA/Tukey HSD post hoc tests. Means bearing different letters superscripts within the same enzyme are significantly different from each other, at a significance level of *p* < 0.05. The value of unfermented control.

Across all tested agro-industrial wastes (pomegranate, mango, grape, and orange pomaces), the unfermented substrates recorded no detectable amylase activity (Figs. 1, 2, 3 and 4). Among the tested microbes, *C. guilliermondii* exhibited the highest amylase activity (4344.67 U/gds) on pomegranate pomace, which was significantly higher than all other microbes within the same pomace and clearly distinct from the unfermented control (*p* < 0.05). This could be due to its metabolic adaptability to the polyphenol-rich nature of pomegranate pomace, together with the fact that the pomegranate pomace has the highest C/N ratio among the tested pomaces (Table 1)^26^. Direct comparison with previous studies is challenging, as this is the first report describing the use of C. *guilliermondii* for amylase production under SSF using pomegranate pomace. However, an α-amylase activity of 2304.19 µmol/L/min was reported by Acourene et al.^27^ upon the submerged fermentation (SmF) of date wastes using *C. guilliermondii* CGL-A10. Multiple studies also reported lower amylase enzymes as summarized in Table 5.

In contrast to amylase, measurable pectinase activity was detected in all unfermented pomaces. Among the tested microorganisms, *C. guilliermondii* again demonstrated superior performance, achieving the highest pectinase activity (4021.57 U/gds) on orange pomace. This value was significantly higher than that observed for other microbes and the unfermented control (*p* < 0.05), confirming the broad enzymatic potential of the strain. Higher pectinase activity was also reported using SmF of orange peel extract using *Aspergillus niger* when the substrate was supplemented with external peptone as a nitrogen source^28^. While Ahmed et al.^29^ and Umsza Guez et al.^30^ reported lower pectinase activities (Table 5).

For xylanase, unfermented mango and grape pomaces displayed detectable activity, whereas pomegranate and orange pomaces did not. As illustrated in Figs. 1, 2 and 3, and 4, *Kluyveromyces marxianus* NRRL Y-8281 exhibited the highest xylanase activity among all tested microorganisms when cultivated on mango pomace (4416.23), showing a statistically significant difference (*p* < 0.05). However, since detectable xylanase activity was also observed in the unfermented mango pomace, xylanase production was not considered for further investigation in this study. Karim et al.^31^ reported lower xylanase yield (2876 U/ml) upon the solid fermentation of mango peel as a substrate using *B. megaterium* (Table 5).

Grape pomace did not support superior glycosidase production, which may be attributed to its relatively low C/N ratio compared to the other pomaces evaluated (Table 1).

Given its remarkable performance across the tested substrates, particularly its high amylase production on pomegranate pomace (4344.67 U/gds), *C. guilliermondii* was selected for subsequent optimization of production parameters. Moreover, amylase activity in the unfermented substrate was negligible compared with the fermented samples, allowing clearer evaluation of microbial enzyme production.

**Table 5:** Summary of recent studies on different glycosidases produced by various fermentation methods using agro-industrial residues as substrates.

Microbial Strain	Substrate	Fermentation Type	Enzyme Produced	Max Enzyme Yield	Reference
*B. licheniformis*	Paddy Straw	SSF	Amylase	1.002 IU/g	^32^
*Geotrichum candidum* PO27	Olive Pomace	SSF	Amylase	180.71 IU/g	^33^
*C. guilliermondii* CGL-A10	Date Wastes	SmF	Amylase	2304.19 µmol/L/min	^27^
*B. subtilis* VSP4	Wheat Bran	SSF	Amylase	169.72 U/gds	^34^
*B. subtilis* J12	Okara Residue	SSF	Amylase	983 U/g	^35^
*Pleurotus pulmonarius*	Cocoa Shells	SSF	Amylase	83.90 U/gds	^36^
*A. oryzae*	Licuri Cake	SmF	Amylase	357.89 U/mL	^37^
*G. candidum* PO27	Olive Pomace	SSF	Amylase	412.94 U/g	^38^
*B. amyloliquefaciens*	Mango Peels	SmF	Amylase	9.03 U/ml	^39^
*A. niger*	Orange Peel Extract	SmF	pectinase	6800 IU/g	^28^
*Penicillium chrysogenum* MF318506	Orange Peel Waste	SmF	pectinase	0.48 U/ml	^29^
*B. megaterium*	Mango Peel	SSF	xylanase	2876 U/mL	^31^
*A. awamori*	Tomato Pomace	SSF	xylanase	100 IU/gds	^30^
*A. awamori*	Tomato Pomace	SSF	pectinase	80 IU/gds	^30^
*Penicillium sp.* FSDE15	Wheat bran	SSF	xylanase	102.34 U/g	^40^

### Optimization of amylase production by *C. guilliermondii* NRRL Y-2075

The superior performance of *C. guilliermondii* on pomegranate pomace prompted further investigation into the operational conditions governing amylase production in this system. A Response Surface Methodology approach was adopted to systematically assess four key fermentation factors.

### Optimization of amylase activity using RSM

Several parameters, including microorganism, substrate selection, pH, temperature, inoculum size, and humidity can greatly affect the SSF process. Optimizing these factors is important for increasing enzyme yield and making the process more cost-effective^41^. In this study, a CCD model was constructed to optimize four key parameters: pH, inoculum size, incubation temperature, and incubation time. The model was used to evaluate the individual and interactive effects of these variables on enzyme production and to determine their optimal conditions, as shown in Table 4.

Table 6 , 7 shows close proximity between actual and predicted amylase yields. The ANOVA results from Table 8 show that the quadratic model was highly significant (*p* < 0.0001) and that all main effects (pH, inoculum size, temperature, and time) and their interactions significantly influence amylase production. The results shown in Table 9 indicate that there is a strong foundation for the model with R², adjusted R², and predicted R².

**Table 6:** CCD results of *C. guilliermondii* amylase.

Run Order	Experimental factors				Actual Value U/gds	Predicted Value U/gds
	A: pH	B: Inoculum size (%)	C: Temperature (°C)	D: Time (h)		
1	7.5	17.5	35	36	3669.17	3671.31
2	5.60798	22.7556	30.7955	24	4672.16	4672.97
3	5.60798	22.7556	39.2045	24	3943.69	3941.77
4	7.5	17.5	35	36	3675.01	3671.31
5	5.60798	12. 2444	39.2045	48	3031.13	3299.71
6	7.5	17.5	35	36	3672.04	3671.31
7	7.5	17.5	35	36	3670.06	3671.31
8	9.39202	12. 2444	39. 2045	24	3769.21	3769.55
9	7.5	17.5	35	36	3667.23	3671.31
10	7.5	17.5	35	36	3674.66	3671.31
11	9.39202	22. 7556	30.7955	24	2991.65	2989.75
12	7.5	17.5	35	36	3670.69	3671.31
13	9.39202	12.2444	30.7955	24	3527.97	3531.53
14	9.39202	22.7556	39.2045	24	3280.9	3283.61
15	5.60798	22.7556	30.7955	24	4673.56	4672.97
16	7.5	17.5	35	36	3674.26	3671.31
17	9.39202	22.7556	30.7955	48	2885.01	2887.18
18	7.5	17.5	35	7.45	4178.77	4178.31

**Table 7:** CCD results of *C. guilliermondii* amylase cont.

19	7.5	17.5	35	36	3675.16	3671.31
20	5. 60,798	12. 2444	39.2045	24	4052.21	4049.80
21	12	17.5	35	36	3092.13	3088.86
22	9. 39,202	22.7556	30.7955	24	2990.1	2989.75
23	5. 60,798	22.7556	30.7955	48	3585.78	3586.50
24	5. 60,798	22.7556	30.7955	48	3589.23	3586.50
25	9. 39,202	22.7556	39.2045	24	3281.78	3283.61
26	9. 39,202	12.2444	39.2045	24	3770.13	3769.55
27	7.5	17.5	35	64.54	3164.01	3164.32
28	9. 392	22.755	30.7955	48	2886.24	2887.18
29	7.5	17.5	25	36	3574.17	3574.94
30	5.60798	22.7556	39.2045	24	3942.11	3941.77
31	5.60798	12.2444	30.7955	48	3433.6	3431.50
32	9.39202	12.2444	30.7955	48	3110.94	3110.10
33	9.39202	12.2444	30.7955	24	3533.59	3531.53
34	5.60798	12.2444	30.7955	48	3430.55	3431.50
35	5.60798	12.2444	39. 2045	48	3300.2	3299.71
36	5.60798	12.2444	30. 7955	24	4835.05	4836.83
37	7.5	17.5	35	36	3675.02	3671.31
38	9.39202	22.7556	39.2045	48	3838.14	3836.29
39	9.39202	12.2444	39.2045	48	4001.32	4003.37
40	7.5	30	35	36	3476.15	3474.53
42	9.39202	12.2444	30.7955	48	3109.24	3110.10
43	9.39202	12.2444	39.2045	48	4002.25	4003.37
44	5.60798	12.2444	39.2045	24	4050.31	4049.80
45	3	17.5	35	36	4251.95	4253.77
46	7.5	5	35	36	3872.13	3868.10
47	5.60798	22.7556	39.2045	48	3510.1	3510.55
48	9.39202	22.7556	39.2045	48	3838.1	3836.29
49	5.60798	22.7556	39.2045	48	3509.1	3510.55
50	5.60798	12.2444	30.7955	24	4837.2	4836.83

**Table 8:** Analysis of variance (ANOVA) for the response surface quadratic model for amylase production.

Source	Sum of Squares	df	Mean Square	F-value	*p*-value	
**Model**	1.054 + 07	10	1.054 + 06	806.19	< 0.0001	significant
A-pH	2.467 + 06	1	2.467 + 06	1886.46	< 0.0001	
B-Inoculum size	2.542 + 05	1	2.542 + 05	194.37	< 0.0001	
C-Temperature	51240.02	1	51240.02	39.18	< 0.0001	
D-Time	2.085 + 06	1	2.085 + 06	1594.43	< 0.0001	
AB	3.380 + 05	1	3.380 + 05	258.38	< 0.0001	
AC	2.240 + 06	1	2.240 + 06	1712.31	< 0.0001	
AD	2.072 + 06	1	2.072 + 06	1584.03	< 0.0001	
BC	15838.00	1	15838.00	12.11	0.0013	
BD	2.489 + 05	1	2.489 + 05	190.29	< 0.0001	
CD	7.721 + 05	1	7.721 + 05	590.34	< 0.0001	
**Residual**	51010.31	39	1307.96			
Lack of Fit	14663.61	14	1047.40	0.7204	0.7354	not significant
Pure Error	36346.69	25	1453.87			
**Cor Total**	1.060 + 07	49				

**Table 9:** Fit statistics of the response surface quadratic model for amylase production.

Std. Dev.	36.17	*R*²	0.9952
Mean	3666.25	Adjusted R²	0.9940
C.V. %	0.9864	Predicted R²	0.9903
		Adeq Precision	115.1729

The close agreement between the predicted and adjusted R² values, together with the high adequate precision, indicated that the model was statistically adequate for exploring the experimental design space. On this basis, the experimental data were described using a second-order polynomial equation, expressed in terms of actual factors, to elucidate the combined effects of the studied variables on amylase activity.

Amylase activity = 21015.64 -1512.72 × pH − 22.64 × Inoculum size − 369.70 × Temperature − 234.57 × Time – 10.33 × pH × Inoculum size + 33.26 × pH × Temperature + 11.21 × pH × Time + 1.01 × Inoculum size × Temperature + 1.40 × Inoculum size × Time + 3.08 × Temperature × Time.

**Fig. 5:**
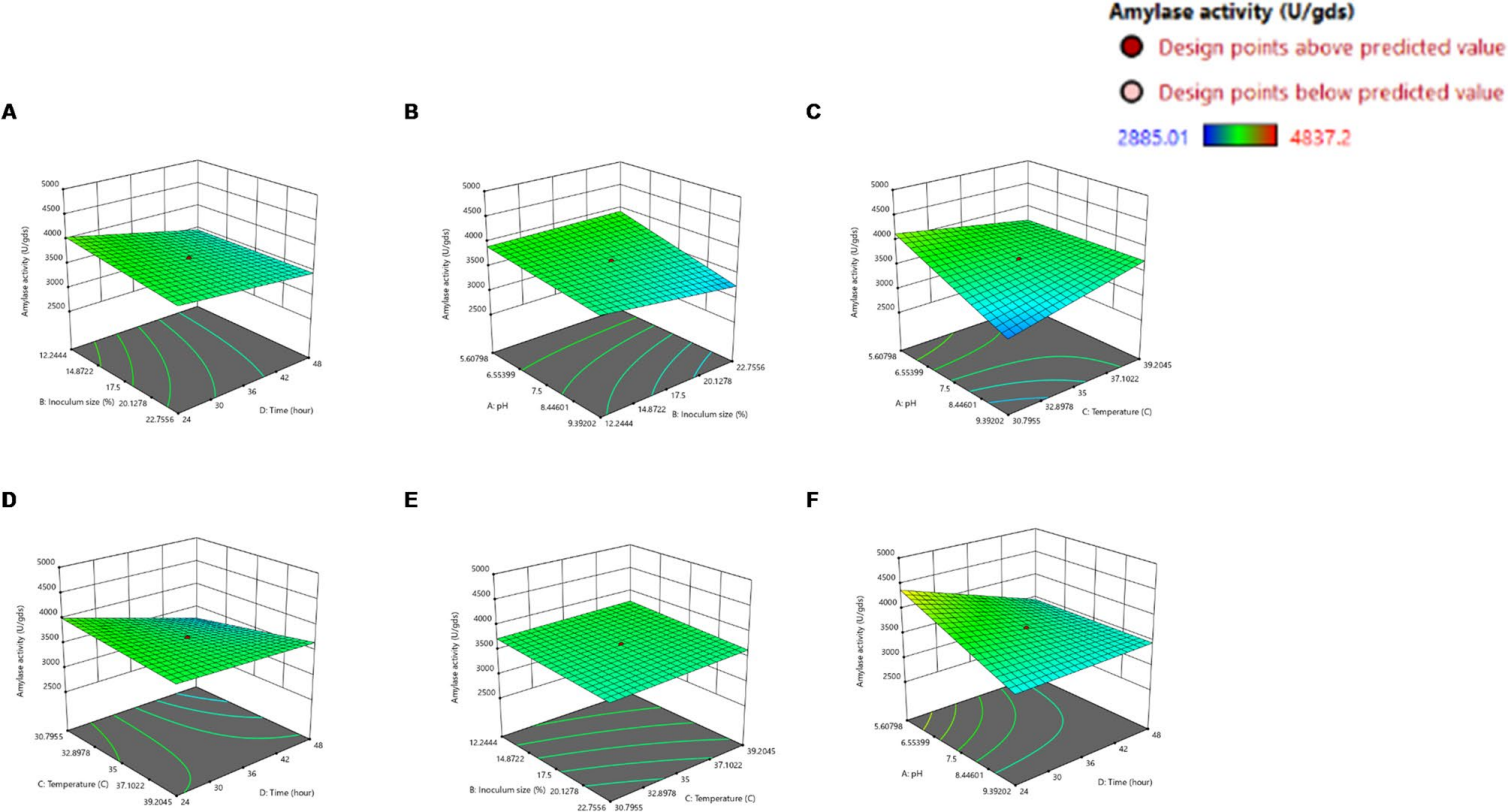
3D response surface plots illustrating the interaction effects of four independent variables on amylase production by *C. guilliermondii* NRRL Y-2075. The plots show the effects of (A) inoculum size (%) and incubation time (h), (**B**) pH and inoculum size (%), (**C**) pH and temperature (°C), (**D**) temperature (°C) and incubation time (h), (**E**) inoculum size (%) and temperature (°C), and (**F**) pH and incubation time (h) on amylase activity, while the other two factors are held constant at their zero (0) level.

The 3D response surface plots as demonstrated in Fig. 5 illustrate the interactive effects of the independent variables on amylase activity under SSF. These visualizations were derived from the CCD model and provide insight into the relationship between variable pairs and their combined influence on enzyme production.

The response surface depicted in Fig. 5 (A) demonstrates that amylase activity increased with increasing inoculum size up to 12.2%, after which any further increase caused a marked decline in enzyme production. This is often attributed to nutrient exhaustion, accumulation of inhibitory metabolites, or degradation of the enzyme itself^42,43^.

In parallel with inoculum effects, Fig. 5 (B) shows that amylase activity increased progressively with rising pH, reaching a maximum at pH 5.6. Further deviation from this optimal pH resulted in a marked decline in enzyme activity. This trend reflects the critical role of medium pH in regulating microbial growth, enzyme biosynthesis, and catalytic stability^42^. Amylases are inherently pH-sensitive and shifts beyond the optimal range can disrupt enzyme conformation, reduce secretion efficiency, and impair substrate–enzyme interactions. Consequently, each amylase-producing microorganism exhibits a defined pH window within which maximal enzyme production and activity are achieved^44^.

Alongside pH regulation, incubation temperature also exerted a pronounced effect on amylase production. According to Fig. 5 (C) raising the incubation temperature to around 30.7 °C markedly enhanced amylase activity, indicating that this temperature is close to the physiological optimum for both microbial growth and enzyme secretion^42^. However, amylase activity decreased sharply at temperatures higher than this optimum, which may be attributed to the thermal stress exerted on the microbe. This behavior is consistent with the reported biochemical characteristics of *C. guilliermondii*, whose optimal growth temperature is around 30 °C, supporting the close linkage between microbial growth physiology and maximal amylase production^45^.

Finally, in terms of process duration, Fig. 5 (D) demonstrates that prolonging the incubation time beyond 24 h led to a significant decrease in the amylase production, this might be coupled with nutrient depletion, accumulation of inhibitory by-products, or enzyme instability over prolonged fermentation periods^42,43^.

Overall, these plots confirm the importance of fine-tuning multiple factors simultaneously rather than optimizing each independently, as significant interactions between variables contribute to maximizing enzymatic output.

**Fig. 6:**
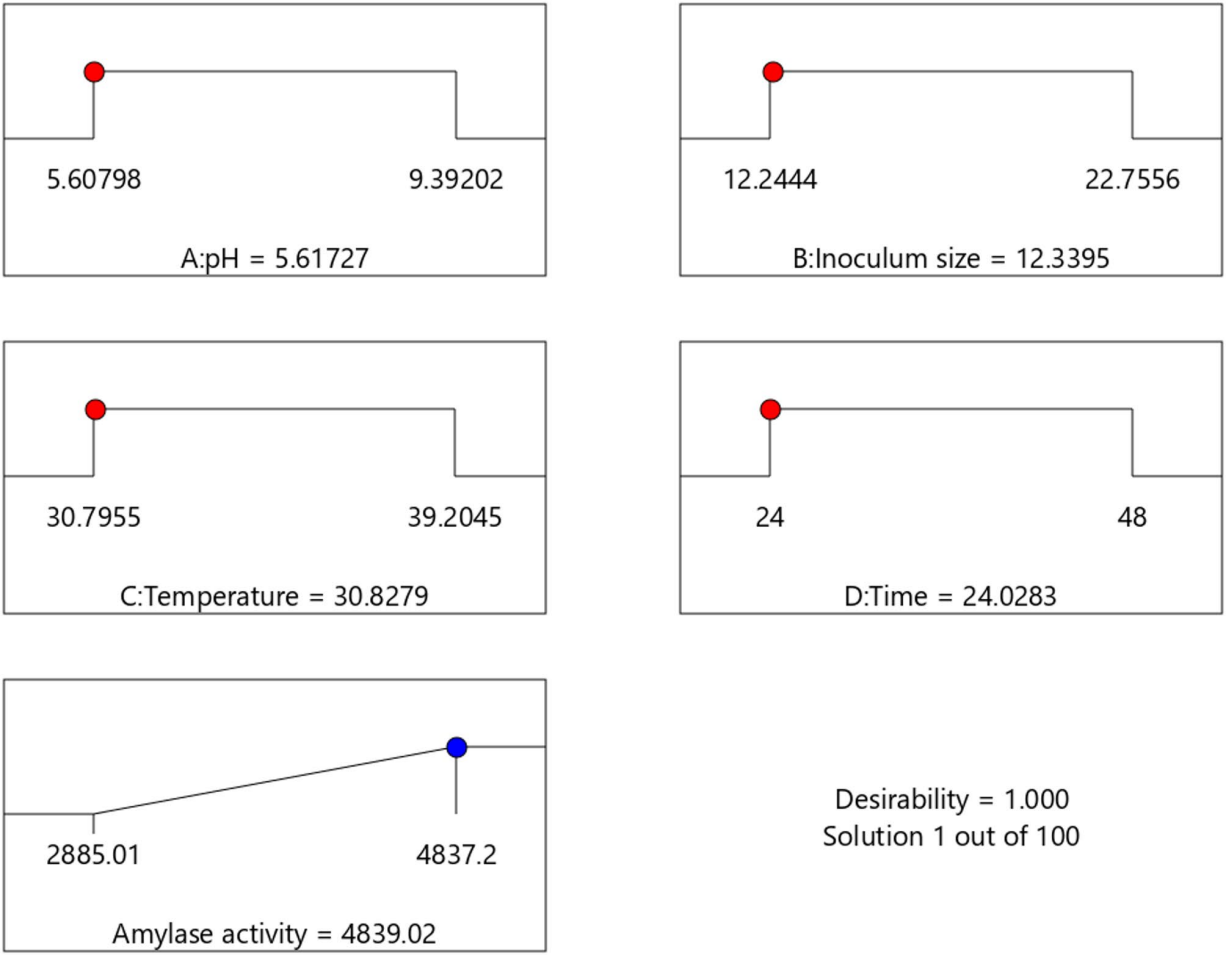
Ramp chart illustrating the numerical optimization of *C. guilliermondii* NRRL Y-2075 amylase production, showing the optimum levels of factors (A) pH, (B) inoculum size, (C) temperature, and (D) incubation time.

As shown in Fig. 6, the optimal fermentation conditions for amylase production by *C. guilliermondii* were pH 5.6, an incubation temperature of 30.7 °C, and an inoculum size of 12.2% (v/w), with peak activity observed after 24 h. These findings are consistent with those of Yalcın et al.^46^, who reported optimal amylase production by *Saccharomycopsis fibuligera* at pH 5.5 and 30 °C on amylase activity medium (AAM), as well as Aggarwal et al.^47^ who reported 30 °C as the optimum temperature for *C. guilliermondii* growth. Likewise, the 24-hour incubation period aligns with the observations of Wanderley et al.^48^ for *Cryptococcus flavus* amylase production on starch-containing medium. The inoculum size also closely matches that reported by Mrudula et al.^49^, who found 15% (v/w) to be optimal for amylase activity by *B. cereus* under solid-state fermentation using wheat bran. Additionally, Acourene et al.^50^ reported comparable optimum conditions for *C. guilliermondii* amylase production from date waste syrup at 30 °C and a pH of 6.0. These findings highlight the rapid enzyme secretion capabilities of certain yeast strains under slightly acidic and mesophilic conditions.

#### Validation of response surface methodology optimum conditions

Validation of the optimized conditions for amylase production confirmed the reliability of the RSM model. The predicted mean amylase activity (4838.05 U/gds) closely matched the experimentally observed value (4839.02 U/gds).

After defining the optimal operational conditions, further experiments were performed to assess whether supplementation with additional nutrients could enhance amylase production or if the selected pomace alone was nutritionally adequate.

### One-factor-at-a-time optimization

**Fig. 7:**
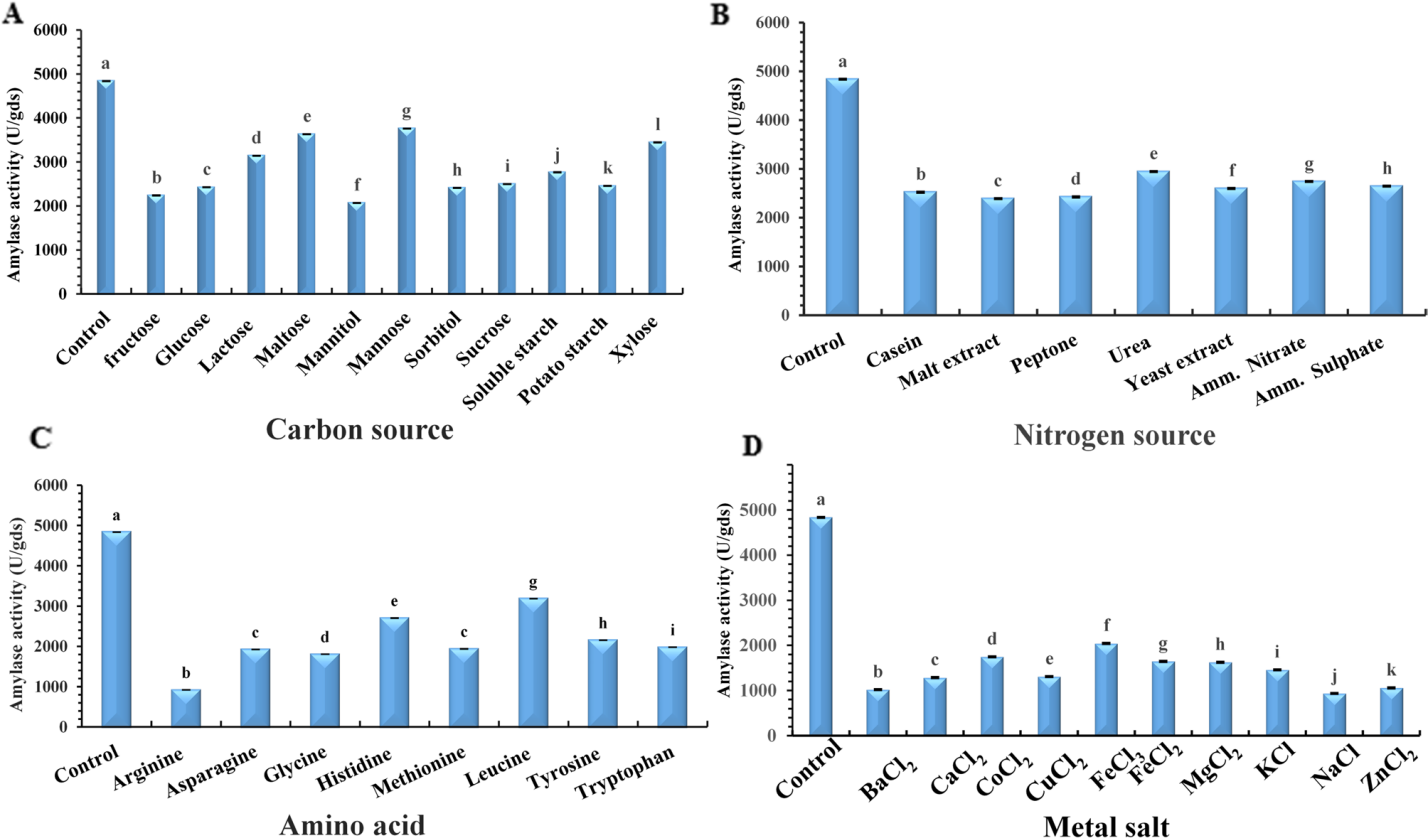
Effect of (**A**) carbon sources, (**B**) nitrogen sources, (**C**) amino acids and (**D**) Metal ions supplemented to pomegranate pomace on production of *C. guilliermondii* NRRL Y-2075 amylase. *Control*: Pomegranate pomace without additives. Different additives were added solely to pomegranate pomace at 1% (w/w). Fermentation was performed under optimal conditions. Error bars indicate the SD from three replicates. Different lowercase letters denote significant differences as determined by one-way ANOVA/Tukey HSD post hoc tests.

Different carbon and nitrogen sources, amino acids and metal ions were added separately to the fermentation medium to study their influence on *C. guilliermondii* amylase production. Statistical analysis (one-way ANOVA, Tukey HSD post hoc, *p* < 0.05) confirmed that the effects of these additives were significant. According to Fig. 7A, the addition of monosaccharides and disaccharides to pomegranate pomace resulted in slightly higher enzyme activity than polysaccharides and sugar alcohols; however, overall amylase production decreased compared to the no-additive control.

Interestingly, the presence of monosaccharides and disaccharides reduced *C. guilliermondii* amylase production, which may be attributed to catabolite repression, as *C. guilliermondii* preferentially assimilates easily metabolised sugars such as glucose, sucrose and galactose over the more complex sugars present in pomegranate pomace, thereby inhibiting the expression of amylase-encoding genes and reducing the amylase production^51,52^. Similarly, the addition of polysaccharides, including starch, also led to reduced enzyme activity. This could be due to the inability of *C. guilliermondii* to utilize starch effectively^47^. This is partially aligned with the findings of Simair et al.^53^ who reported supplementation of the medium with agro-industrial waste, specifically molasses and date syrup, to have higher amylase production than starch addition, but contrary to Saad et al.^54^, who reported enhanced amylase production by *B. licheniformis* cultivated in a modified starch broth medium. Similarly, the addition of sugar alcohols significantly suppressed amylase production in this study, which contradicts the findings of Saha et al.^55^, who reported an increased amylase yield by *B. amyloliquefaciens* when cultivated on wheat bran under SSF upon the addition of inositol and mannitol.

Optimal conditions for maximum enzyme production can vary considerably depending on the specific microbial strain^56^. The type and concentration of nitrogen sources play a crucial role in regulating enzyme synthesis by influencing both nitrogen assimilation and metabolic activity^57^. As shown in Fig. 7B, the addition of external nitrogen sources generally resulted in a reduction in amylase production. However, urea caused the least reduction, yielding 2944.77 U/gds, compared to the highest activity observed in the no-additive control (4838.23 U/gds). This result aligns with the findings of Singh, Ravi et al.^58^ who reported that the addition of various organic and inorganic nitrogen sources led to a decrease in α-amylase production by *B. cereus* MTCC 1305 under SSF using wheat bran as a substrate.

Amino compounds are considered stimulators of amylase synthesis and excretion rather than primary nitrogen or carbon sources^59^. However, in the present study, the addition of amino acids to pomegranate pomace resulted in a significant decline in amylase production by *C. guilliermondii*. As illustrated in Fig. 7C, leucine supplementation caused a moderate decrease in enzyme yield, while arginine exhibited a strong inhibitory effect. These findings partially align with those of Chaurasia et al.^60^, who reported that asparagine supplementation enhanced *Rhizopus oryzae* amylase production using Fernando’s broth medium, whereas the addition of isoleucine, phenylalanine, and lysine inhibited its synthesis.

The addition of metal ions to the fermentation medium negatively affected amylase production by *C. guilliermondii*. As presented in Fig. 7D, Fe⁺³ caused the least inhibition with 2044.23 U/gds, compared to the no additive control, while Na⁺ caused the greatest suppression (935.7 U/gds), which may be attributed to the fact that NaCl exerts osmotic pressure on *C. guilliermondii* cells^61^. These results contradict previous studies. For instance, Abo-Kamer et al.^56^ reported Ca⁺² and Mg⁺² to enhance amylase production in *B. cereus* A1-5 using synthetic starch medium under submerged fermentation, whereas Ba⁺² had an inhibitory effect. Similarly, Rehman et al.^62^ found that Ca^+ 2^ and Na^+^ enhanced amylase production in *B. cereus* AS2 using Luria basal broth medium, while Mg^+ 2^, Zn^+ 2^, Hg^+ 2^, Cu^+ 2^ and Fe^+ 3^ were inhibitory. The reduced amylase production by *C. guilliermondii* upon the addition of external carbon, nitrogen sources, amino acids, and metal ions highlights the inherent nutritional adequacy of pomegranate pomace. With a composition of 4.9% protein, 17.7% carbohydrates, and a variety of essential minerals including calcium, potassium, phosphorus, sodium, iron, copper, manganese, and zinc the pomace alone appears sufficient to support microbial growth and enzyme synthesis^26^. The introduction of external additives may have disrupted this natural nutrient balance, resulting in inhibitory rather than beneficial effects on enzyme product.

## Conclusion

This study indicates that agro-industrial fruit wastes, especially pomegranate pomace, are promising, consistent and sustainable substrates for microbial enzyme production under solid-state fermentation. *C. guilliermondii* NRRL Y-2075 was the most effective strain for amylase production with a maximum activity of 4344.67 U/gds. Using RSM, we determined optimum fermentation conditions and experimentally validated these results with a maximum amylase production at 4838.23 U/gds. The small difference between predicted and observed values indicates that the model is reliable. External supplementation of various additives (carbon, nitrogen sources, amino acids or metal ions) to the fermentation medium indicated an enzyme activity reduction, further establishing the nutritional value of simply using pomegranate pomace. This study provides valuable information toward developing cost-effective, environmentally friendly options for enzyme production while also promoting the possibilities of exploiting agro-waste as a substrate for sustainable industrial biotechnology. Future optimization studies should incorporate key physical parameters such as moisture content, aeration, and particle size to further improve enzyme production under SSF conditions.

## Data Availability

The datasets generated and/or analysed during the current study are available in the NCBI GenBank repository under the accession numbers LC315566 (https://www.ncbi.nlm.nih.gov/nuccore/LC315566), LC315565 (https://www.ncbi.nlm.nih.gov/nuccore/LC315565), LC315920 (https://www.ncbi.nlm.nih.gov/nuccore/LC315920), LC438914 (https://www.ncbi.nlm.nih.gov/nuccore/LC438914), PV569636 (https://www.ncbi.nlm.nih.gov/nuccore/PV569636), PV569637 (https://www.ncbi.nlm.nih.gov/nuccore/PV569637), PV569638 (https://www.ncbi.nlm.nih.gov/nuccore/PV569638), PV569639 (https://www.ncbi.nlm.nih.gov/nuccore/PV569639), PV569640 (https://www.ncbi.nlm.nih.gov/nuccore/PV569640) and PV569641 (https://www.ncbi.nlm.nih.gov/nuccore/PV569641).

## Abbreviations

AOAC: Association of Official Analytical Chemists
ANOVA: Analysis of Variance
C/N ratio: Carbon/Nitrogen ratio
CCD: Central Composite Design
Df: Degrees of Freedom
GHs: Glycoside hydrolases
h: Hour(s)
min: Minute(s)
OFAT: One Factor at a Time
RSM: Response Surface Methodology
SD: Standard Deviation
SSF: Solid State Fermentation
U/gds: Units per gram dry substrate
